# A Bridge Crack Segmentation Algorithm Based on Fuzzy C-Means Clustering and Feature Fusion

**DOI:** 10.3390/s25144399

**Published:** 2025-07-14

**Authors:** Yadong Yao, Yurui Zhang, Zai Liu, Heming Yuan

**Affiliations:** 1 Institute of Transportation, Inner Mongolia University, Hohhot 010070, China; 311986442@imu.edu.cn (Y.Y.); 32324048@mail.imu.edu.cn (Y.Z.); 32324005@mail.imu.edu.cn (Z.L.); 2 Inner Mongolia Engineering Research Center of Testing and Strengthening for Bridges, Inner Mongolia University, Hohhot 010020, China

**Keywords:** fuzzy C-means clustering, bridge crack detection, multi-feature fusion, connected domain labeling, circularity threshold, unsupervised detection

## Abstract

In response to the limitations of traditional image processing algorithms, such as high noise sensitivity and threshold dependency in bridge crack detection, and the extensive labeled data requirements of deep learning methods, this study proposes a novel crack segmentation algorithm based on fuzzy C-means (FCM) clustering and multi-feature fusion. A three-dimensional feature space is constructed using B-channel pixels and fuzzy clustering with c = 3, justified by the distinct distribution patterns of these three regions in the image, enabling effective preliminary segmentation. To enhance accuracy, connected domain labeling combined with a circularity threshold is introduced to differentiate linear cracks from granular noise. Furthermore, a 5 × 5 neighborhood search strategy, based on crack pixel amplitude, is designed to restore the continuity of fragmented cracks. Experimental results on the Concrete Crack and SDNET2018 datasets demonstrate that the proposed algorithm achieves an accuracy of 0.885 and a recall rate of 0.891, outperforming DeepLabv3+ by 4.2%. Notably, with a processing time of only 0.8 s per image, the algorithm balances high accuracy with real-time efficiency, effectively addressing challenges, such as missed fine cracks and misjudged broken cracks in noisy environments by integrating geometric features and pixel distribution characteristics. This study provides an efficient unsupervised solution for bridge damage detection.

## 1. Introduction

In recent years, computer-based image processing technology has been widely applied in the field of bridge damage identification. This advancement is closely tied to the evolution of sensor technologies, particularly high-resolution optical sensors deployed on unmanned aerial vehicles (UAVs). Specifically, the technology of using drones equipped with multi-spectral cameras and inertial measurement units (IMUs) to capture the damaged parts of bridges and conduct damage assessments is becoming increasingly mature. Among them, automatic crack detection (ACD), as one of the typical technologies of computer V = vision (CV), has become a research hotspot in this field. Currently, research on ACD technology mainly focuses on the following two directions [1]: one is image processing technology (IP) based on traditional feature recognition algorithms; and the second is damage recognition technology based on deep learning (DL) [2]. Traditional image processing techniques typically rely on specific algorithms, such as edge detection, clustering analysis, threshold segmentation, and connected domain labeling, utilizing manually designed features for crack detection. The technology based on deep learning simulates the neural network model of human neuron architecture to achieve automatic classification and the recognition of damage types and has been widely used in bridge crack detection tasks [3]. Table 1 shows the main directions and classification framework of current ACD research.

Although traditional feature recognition algorithms (such as first denoising through Gaussian filtering and then using edge detection algorithms to extract the edges of each element in the image) can accurately detect the location of pixel gradient mutations; that is, the edges of objects in the image and their ability to automatically and accurately identify crack positions are significantly limited by the noise interference caused by concrete spalling. To address this issue, related research has proposed the connected domain labeling method to further improve the accuracy and robustness of crack detection [11]. This method eliminates noise interference by setting a threshold, but the value of the threshold needs to be determined based on the size of the noise [12]. This results in such methods typically only being effective on specific sizes of noise and relying on the matching relationship between the original image size and the noise size. Although this method has shown some effectiveness in crack identification, it still has significant shortcomings and limitations. When dealing with complex and changing environmental factors in practical scenarios, its reliability and applicability may be significantly affected [13].

Compared to traditional feature recognition algorithms, deep learning-based methods can utilize more and deeper convolutional layers, theoretically capable of handling more complex interference situations [14]. At present, various deep learning algorithms have performed well in detecting concrete characteristic defects. However, these methods often rely on supervised learning and require a large amount of manually annotated data input, especially in visual data annotation. This high demand for annotated data significantly limits its scalability and efficiency. In actual bridge damage detection, the raw images obtained are often not annotated, and annotating the dataset not only consumes a lot of time but may also introduce the subjective bias of the annotator [10].

Given the features of current algorithms, this paper proposes a novel algorithm for crack identification. It is based on fuzzy C-means (FCM) clustering and fuses multiple features. The algorithm addresses the limitation of traditional feature identification methods that require manual threshold adjustment for noise reduction. Additionally, it eliminates the need for extensive data labeling and enables automatic crack identification in images.

## 2. Method

### 2.1. Fuzzy c-Means Clustering

**Figure 1:**
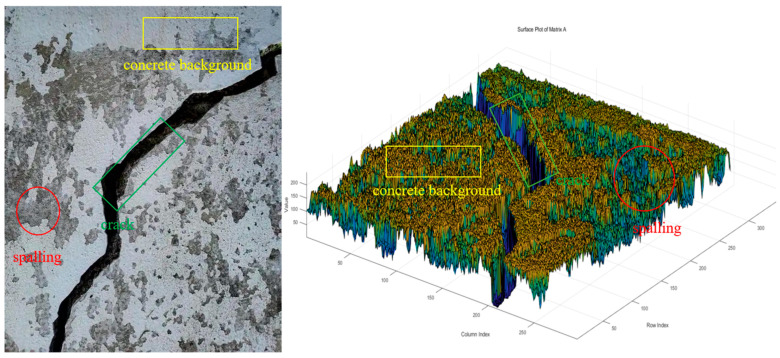
Comparative 3D data visualization of crack characteristics.

Taking the crack images with severe concrete spalling as an example (as shown in Figure 1), this study conducted an analytical investigation by constructing a three-dimensional data plot using pixel intensity values as the z-axis. The analysis of original bridge crack images revealed distinct distribution patterns among the following three characteristic regions: the concrete background, concrete spalling areas, and primary crack zones. Specifically manifested through there is a significant color difference between cracks and the surrounding concrete environment. Specifically, under appropriate lighting conditions, cracks typically appear black, forming a distinct color contrast with the surrounding light gray concrete background. At the same time, although the slight peeling of concrete and the color of aggregates are different from the color of cracks, their color tone is relatively dull compared to the overall concrete background. Therefore, when clustering based on pixel values as reference values, the number of clusters can be set to 3 categories, namely concrete background, concrete spalling, and crack body.

In the RGB color space, the blue component (B) value in the crack area is usually higher, presenting a darker hue. If the crack is not filled with moisture or other substances, its color may be close to black or dark gray, indicating relatively low values for red (R) and green (G). Therefore, selecting the blue component (B) in RGB as the representative value helps simplify the calculation process [4].

In this study, the pixel values of channel B in the image were used as data for fuzzy C-means clustering analysis. The fuzzy C-means (FCM) clustering algorithm, initially proposed by Dunn in 1973 [15] and systematically refined by Bezdek in 1981 [16], addresses the limitations of traditional K-means algorithms—such as sensitivity to initial cluster centers and rigid data partitioning—by introducing membership functions to quantify the degree of data points belonging to multiple clusters [15,16]. The core of the classical FCM lies in minimizing the objective function defined as the weighted sum of squared distances between data points and cluster centers, controlled by a fuzziness parameter m (typically set to 2) to regulate the smoothness of membership degrees [7,17]. In fuzzy set theory, the membership degree of an element can take any value between 0 and 1, indicating the degree to which the element belongs to a certain set. This means that different pixel values are not forcibly classified into a specific category but are allocated among multiple categories based on their membership degree. The membership degree of pixel values after FCM clustering exhibits a smooth transition characteristic [16], which provides a solid foundation for subsequent algorithm steps.

Early studies validated FCM’s effectiveness in image segmentation and pattern recognition but highlighted challenges, such as susceptibility to local optima and high computational complexity [7,15].

To overcome these limitations, researchers have proposed various enhancements, as follows: Genetic algorithm integration: Xu et al. (2007) [9] combined genetic algorithms with FCM to optimize initial cluster centers through global search, effectively mitigating local optima. Particle swarm optimization (PSO): Ye et al. (2019) [7]. utilized PSO to accelerate convergence, demonstrating superior robustness in complex datasets (e.g., concrete defect images) [7,16]. Multi-space collaborative clustering: Chen et al. (2020) [6] introduced a multi-feature fusion strategy by integrating RGB and Lab color space features into FCM. This approach, coupled with weighted membership fusion, achieved an accuracy of 89.5% in concrete spalling detection [6]. These advancements underscore FCM’s adaptability in handling overlapping and ambiguous data structures, while balancing computational efficiency and clustering precision.

Based on an analysis of crack images, this study proposes a method grounded in the fuzzy C-means (FCM) algorithm. By leveraging the pixel distribution patterns of crack images, the distribution of primary crack pixels is defined to achieve the segmentation of cracks from noise and concrete backgrounds.

The operation process of FCM can be divided into the following key steps [9]:

1. Select the number of clusters (c): Specify the number of clusters to be formed. In general, the application of the fuzzy C-means method in crack detection faces several challenges, particularly the selection of the optimal number of clusters CCC. This parameter is crucial, as improper tuning can lead to the over-segmentation or under-segmentation of the image. Too many clusters may introduce noise and confuse cracks with other textures, while too few may merge distinct regions, thus compromising detection accuracy.

In light of prior analysis on severely degraded concrete spalling crack images, the cluster number was provisionally defined as 3 (cracks, concrete spalling noise, and concrete background) to balance computational efficiency and segmentation accuracy. The rationality of FCM cluster number being 3 will be verified in Section 3.

2. Initialize the membership matrix (u):

FCM uses a membership matrix u. Among them, $u_{ij}$ represents the membership degree of the data point belonging to cluster c. The membership degree indicates that the data point belongs to the cluster. The sum of membership degrees is 1, as follows:

(1) $$\sum_{i=1}^{c}u_{ij}=1$$

Among them, c represents the number of clusters, and j represents the index of data points.

3. Objective function:

The goal of FCM is to minimize the following objective function:

(2) $$J\left(U,V\right)=\sum_{i=1}^{c}\sum_{j=1}^{n}u_{ij}^{m}{∥x_{j}-v_{i}∥}^{2}$$

Among them:

n is the number of data points.

$v_{i}$ is the center of cluster $C_{i}$.

m is the fuzziness index (usually set to 2), which controls the fuzziness of membership degrees.

4. Calculate membership degree:

Calculate the membership degree of each data point based on the current clustering center, as follows:

(3) $$U_{ij}=\frac{1}{{\sum_{k=1}^{c}\left(\frac{\left\|x_{j}-v_{i}\right\|}{\left\|x_{j}-v_{k}\right\|}\right)}_{\frac{2}{m-1}}}$$

5. Update clustering centers:

Update the center $\nu_{i}$ of each cluster based on the current membership matrix u.

(4) $$\nu_{i}=\frac{\sum_{i=1}^{n}U_{ij}^{m}X_{j}}{\sum_{j=1}^{n}U_{ij}^{m}}$$

This formula indicates that the new cluster center is the weighted sum of all data points, with a weight equal to the power of membership degree m.

6. Iteration:

By setting the number of iterations, repeat steps 2 and 3 until the stopping condition is met, that is, the objective function takes the minimum value. Here, the number of iterations is set to 100, and the convergence accuracy is 1.0 × 10^−6^.

### 2.2. Preliminary Denoising Using Fuzzy C-Means Clustering

After FCM clustering analysis, the original crack images were divided into the following three categories: one is the concrete background pixels, another is the peeling pixels around the concrete cracks, and the last is the main pixels of the concrete cracks. Considering that the RGB values corresponding to the black pixels of concrete cracks in the original image are (0,0,0), we adopted the strategy of reversing pixel values. Specifically, in the newly generated layer, black pixels are marked as blue (with RGB values of (0,0,255)) for further analysis. In the selection of original image samples, representative crack specimens were chosen from the dataset based on morphological diversity and contextual relevance. Specifically:

Type A exhibits large-area concrete spalling along the crack propagation path, demonstrating structural degradation severity.

Type B contains intersecting cracks with angular discontinuities, reflecting complex stress distribution patterns.

Type C features distinctive surface imprints co-localized with primary crack trajectories, accompanied by observable water stains in adjacent regions, indicative of environmental interaction effects.

**Figure 2:**
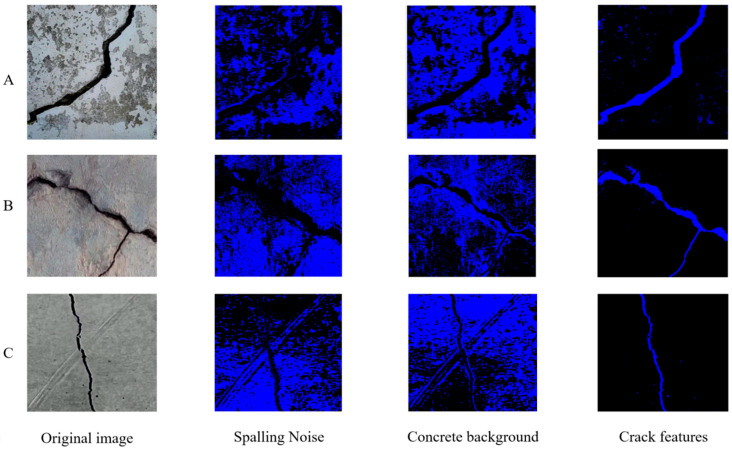
Fuzzy C-means clustering result chart.

This ensures the comprehensive coverage of typical crack manifestations in concrete infrastructure, addressing both intrinsic material failure modes (Types A–B) and extrinsic deterioration factors (Type C). The selection criteria align with guidelines for structural defect characterization, prioritizing diagnostically significant morphological attributes. Figure 2 shows the image effect after pixel inversion processing.

**Figure 3:**
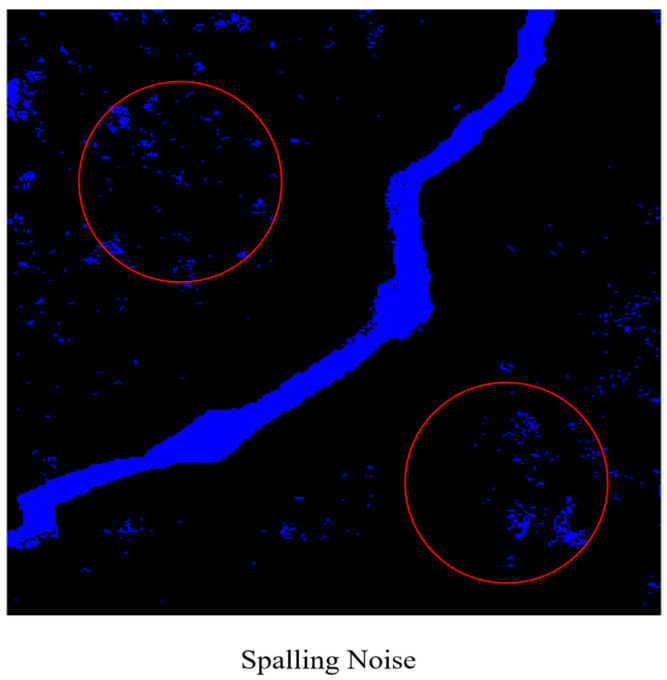
Spalling noise.

After the first-step FCM clustering, the crack main body as well as the noise with pixel values close to black (which cannot be effectively segmented by clustering) can be separated from the concrete background and weak noise. However, it is evident that FCM is helpless in dealing with stubborn noise, as shown in Figure 3. To address this, we analyzed 200 crack and noise samples, fitting their circularity distributions with Gaussian models, which provides a new approach to tackle the issue.

### 2.3. Denoising with Connected Component Labelling and Circularity

After the initial segmentation of concrete crack images using the FCM algorithm, some stubborn noise still exists. To obtain more accurate segmentation results, a post-processing method based on 8-connected neighborhood connected component labeling and circularity-based denoising is utilized. The 8-connected rule can more comprehensively consider the connections between pixels, making the division of connected regions more in line with the actual distribution characteristics of cracks. This is especially suitable for situations where cracks may have complex directions and branches [17,18].

**Figure 4:**
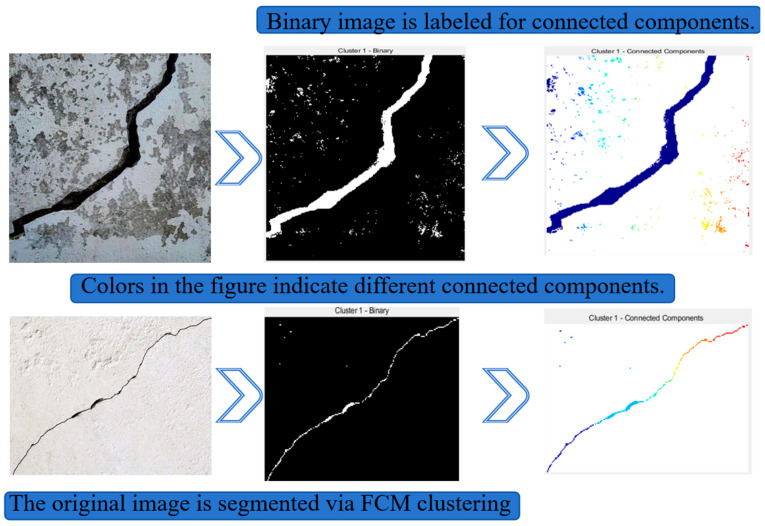
Connected component labeling results.

Firstly, the connected component labelling algorithm is applied to the segmented image. This algorithm identifies and labels each connected region in the image, which helps in distinguishing the crack regions from the noise. Each pixel in the image is traversed to find connected regions that meet certain connectivity criteria, and each connected region is assigned a unique label. The connected component labelling results are shown in Figure 4. Figure 4 includes a concrete crack image with severe spalling (top) and a small-size crack image (bottom).

Subsequently, calculate the circularity of each connected region. Circularity is an indicator quantifying how closely a region’s shape resembles a circle. The rationale is that real cracks typically manifest as irregular lines, whereas noise often appears as near-circular particles. Thus, circularity is introduced for noise removal. Its mathematical expression is as follows:

(5) $$\phi =\frac{4\pi A}{S^{2}}$$

**Figure 5:**
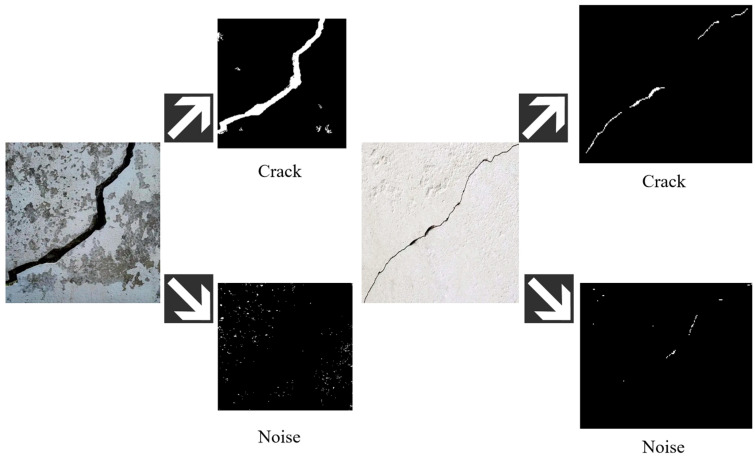
Denoising effect image.

Among them, ϕ denotes the circularity, where S represents the perimeter of the marked connected region, and A is the pixel area within the connected region. The noise removal effect is illustrated in Figure 5. Set the circularity threshold at less than 0.3 for cracks and greater than 0.8 for noise and observe the noise-reduction effect of this method.

Despite its effectiveness in noise removal to some extent, the performance of circularity as a feature is highly dependent on the rationality of threshold selection. Traditional fixed-threshold methods are confronted with the following two major challenges: (1) they lack the ability to distinguish between cracks and noise that are morphologically similar, as shown in the upper side of Figure 5; (2) they perform poorly in denoising images with small-sized and intermittent cracks, as illustrated on the lower side of Figure 5.

### 2.4. Crack Identification Based on Crack Pixel Characteristics

**Figure 6:**
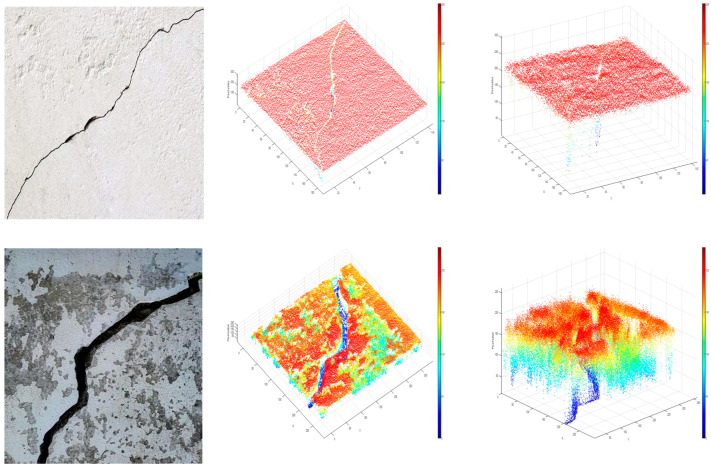
Original image and Crack three-dimensional data graph.

When analyzing the pixel value distribution of the main image of the crack, the row and column positions of each pixel in the image can be regarded as coordinates in a two-dimensional coordinate system, and the pixel value of that pixel corresponds to its height value in three-dimensional space. Through this transformation, we can gain a deeper understanding of the structural characteristics and spatial distribution of cracks and lay a data foundation for subsequent image processing and damage analysis. As shown in Figure 6, the three-dimensional data demonstrates the specific implementation of this process.

**Figure 7:**
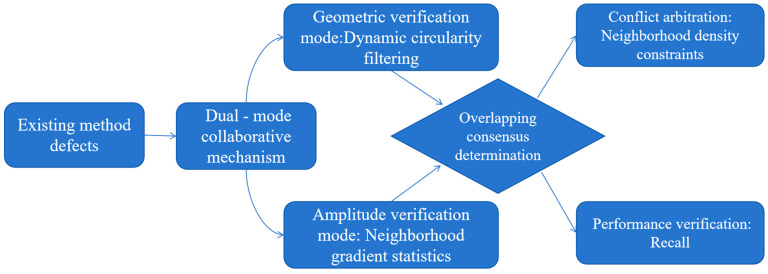
The flowchart of the algorithm.

In light of the above characteristics, this study aims to address the limitations of traditional circularity-based methods in crack image analysis. By leveraging pixel-based features to identify cracks, the proposed approach seeks to overcome these limitations. Specifically, it involves adaptively adjusting the roundness threshold and implementing a dual-criterion assessment of the crack’s main body position. The workflow of our algorithm is as below: Identify principal crack pixels ⟶ Neighborhood search ⟶ Connected-region labeling and circularity computation ⟶ Pixel-count matching ⟶ Output. This process is visually illustrated in Figure 7.

Among them, GVM (geometric verification mode) refers to the following:

When analyzing crack images using circularity theory, we start at 0.1 and increment by 0.1 to screen connected regions with circularity between 0 and 1. This is based on the mathematical definition that the circularity of connected regions after labeling is greater than 0 and ≤1. Extensive experiments show a 0.1 increment balances computational efficiency and screening accuracy. It avoids excessive steps from a smaller increment and missing potential crack features from a larger one. Thus, we set the circularity threshold at 0.1 to identify the main crack regions in the image. The rationale for designating the pixel intensity values within this principal crack body as the main pixel intensities of the crack body is as follows. Initially, the FCM clustering technique was employed to perform preliminary noise reduction on the image, which effectively constrained the pixel intensity values within a specific range. Subsequently, by further denoising using a roundness threshold of 0.1, the remaining structures are the slenderest and most elongated principal bodies of the cracks. Thus, it is reasonable to consider the pixel intensities within these principal crack bodies as the main crack intensities.

However, using a small roundness threshold may result in the loss of small and intermittent cracks.

**Figure 8:**
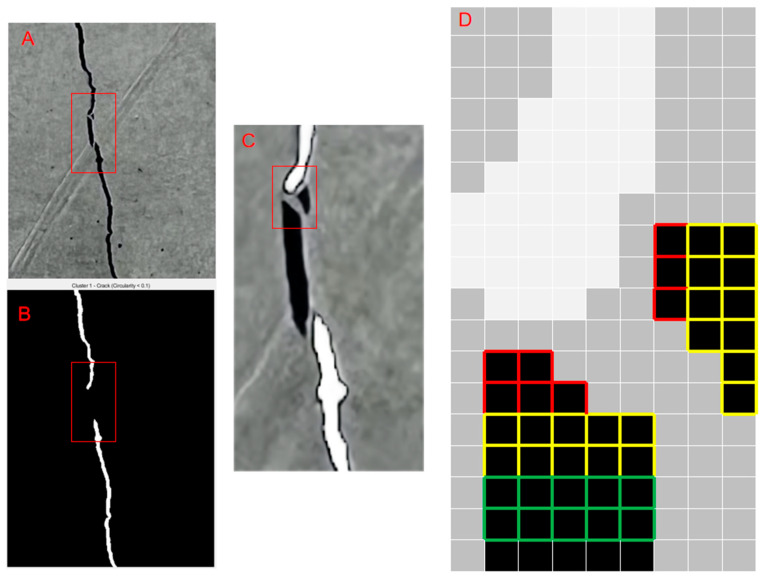
Crack detection and neighborhood search process in concrete surfaces.

Therefore, we introduce the AVM, where the specific approach is as follows:

We can perform a neighborhood search on the original image based on the range of crack pixel amplitudes. This helps to compensate for the small and intermittent cracks that are identified as noise due to geometric features. The neighborhood search process is also shown in Figure 8. Figure 8A is the original image. Figure 8B shows the primary crack image with a circularity of 0.1. Figure 8C illustrates the neighborhood search starting from the main crack pixels in the original image. In Figure 8D, red pixels denote the first-searched pixels. Yellow pixels are newly searched based on red ones. Green pixels are newly searched based on yellow ones. The search is conducted within a 5 × 5 neighborhood, targeting points that fall within the pixel intensity range characteristic of typical cracks.

The pixel amplitude range is defined as follows:

Crack pixel set: after FCM clustering, the cluster with the smallest pixel values is denoted as $C_{{crack}_{t}}$, and its pixel value set is $\left\{x_{1},x_{2},\ldots ,x_{n}\right\}$.

The amplitude range is defined using the mean and standard deviation to establish the search range:

(6) $$Range=\left[\mu -k\sigma ,\mu +k\sigma \right]$$

where

$\mu =\frac{1}{n}{\sum_{i=1}^{n}X}_{i}$: represents the mean value of the crack pixel set.

$\sigma =\sqrt{\frac{1}{n}}{\sum_{i=1}^{n}\left(X_{i}-\mu \right)}^{2}$: represents the standard deviation of the crack pixel set.

k is the expansion coefficient, experimentally set to 2 to cover approximately 95% of the pixels.

Neighborhood search rules:

For each crack pixel, define its neighborhood (as shown in Figure 8C,D).

For pixels within the defined neighborhood $q\in N\left(p\right)$, if they meet the following conditions:

(7) $$X_{q}\in \left[\mu -k\sigma ,\mu +k\sigma \right]\mathrm{and}\;q\notin C_{crack}$$

Mark q as a new crack pixel and add it to the search queue.

Once the search is completed, we count the number of pixels in the crack body. Then, we compare it with the number of pixels in crack images identified as cracks under different roundness thresholds with a step size of 0.1.

When the two modes’ detection results conflict (e.g., the geometric mode identifies a crack but the magnitude mode rejects it), an arbitration mechanism based on morphological constraints is triggered. If the 8-neighborhood density of $X_{i}$ exceeds 60%, $X_{i}$ is included in the final segmentation set.

By using the same number of pixels as the matching criterion, we can finally output the overlapping part of the two identification modes as the segmented image of the crack body.

**Figure 9:**
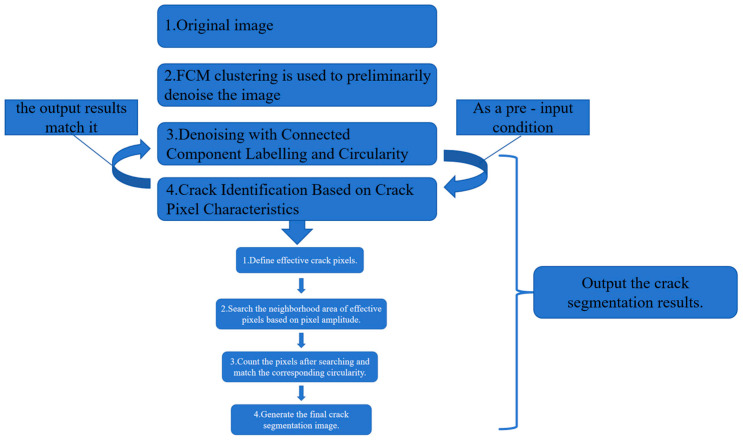
A bridge crack detection algorithm based on FCM with feature fusion.

In summary, this paper proposes a bridge crack detection algorithm based on FCM with feature fusion. The overall process and principle are shown in Figure 9.

**Figure 10:**
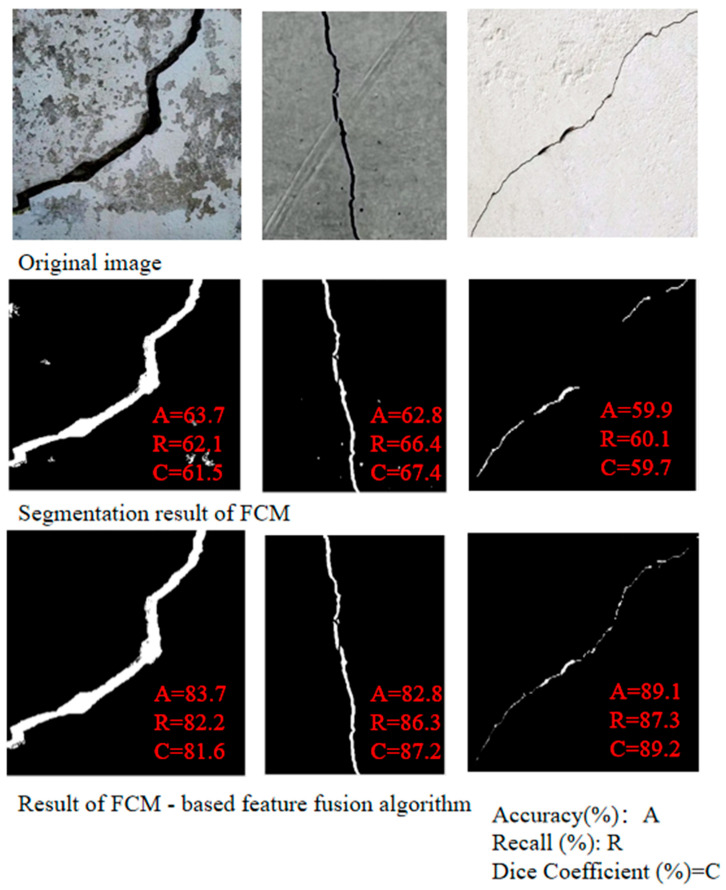
Comparison of crack segmentation results using different algorithms based on FCM.

The preliminary verification based on the above images and algorithms shows that the FCM algorithm combined with circularity and the FCM-based feature fusion algorithm can enhance crack segmentation under the set parameters, as depicted in Figure 10.

## 3. Experimental Demonstration

Traditional segmentation algorithms struggle with complex scenarios like blurred crack edges and noise. This study focuses on the fuzzy C-means (FCM) algorithm. It compares FCM’s performance with hard-clustering algorithms (C-means, K-means) and density-based algorithms (DBSCAN). The aim is to offer a more robust segmentation solution for feature fusion-based crack detection using FCM.

### 3.1. Dataset Description

To verify the rationality of FCM parameter selection and the performance differences between our algorithm and others in crack identification, we utilized the Concrete Crack images for segmentation dataset and the SDNET2018 dataset. The Concrete Crack images for segmentation dataset, created and published in 2022 by Cai et al. [19]. (https://data.mendeley.com/datasets/p86fm2h39s (accessed on 4 May 2025)), consist of around 740 training images, 246 testing images, and 246 validation images, all of varying sizes and accompanied by corresponding labels. The SDNET2018 dataset, constructed in 2018 by Maguire, Marc, Dorafshan, and Sattar Thomas, Robert J, and released for concrete crack detection research, contains over 56,000 images of cracked and uncracked concrete surfaces, including bridge decks, walls, and roads. The crack widths in this dataset range from 0.06 mm to 25 mm, and it also includes images of various obstacles, such as shadows and surface roughness [20]. During the dataset construction process, all original images were first converted from RGB to grayscale and normalized to the [0, 1] range before being resized to 256 × 256 to enhance model training stability. The labels for the Concrete Crack images for the segmentation dataset were manually annotated by professional engineers, defining cracks as continuous linear regions with a width of over 0.1 mm, while non-crack regions include concrete backgrounds and stains. Finally, we combined all images from the Concrete Crack images for segmentation dataset with 1000 randomly selected images from the SDNET2018 dataset. The combined dataset was then divided into training, validation, and testing sets in a 7:2:1 ratio for subsequent model training and performance evaluation. The dataset includes various concrete and traditional stone structures, ensuring training diversity and model generalization. We also statistically analyzed the crack width distribution and noise types in the two datasets. The training, validation, and test sets maintain a good balance in crack size and noise categories, thus avoiding data bias in model performance evaluation.

**Figure 11:**
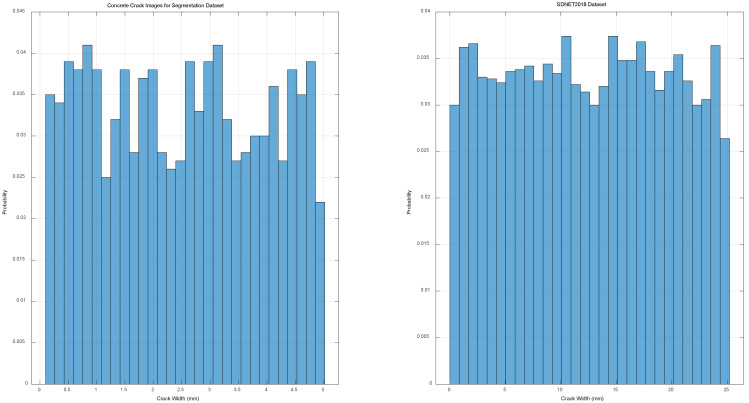

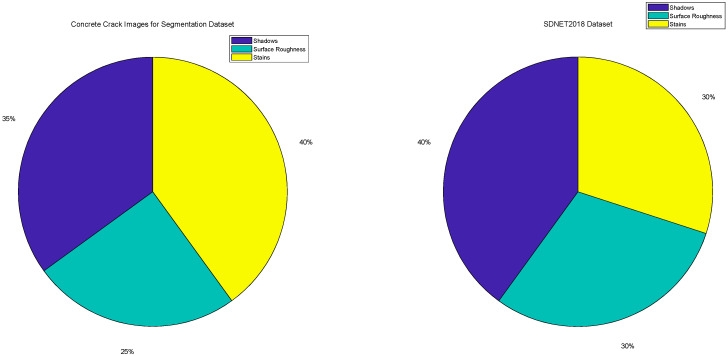
Distributions of crack width and noise type in the datasets.

The specific distributions of crack width and noise type within the datasets are illustrated in Figure 11.

### 3.2. Comparison of Different Clustering Algorithms

This study conducted the following experiments to evaluate the crack segmentation quality of different algorithms by controlling their respective parameters:

1. C-Means Algorithm: The key parameter is the number of cluster centers. After numerous experiments, it was found that setting the number of cluster centers to 3 effectively distinguishes cracks, backgrounds, and other interfering objects (e.g., stains, textures), ensuring accurate crack segmentation. While increasing the number of cluster centers sometimes allows for more detailed local distinctions in complex images, the overall improvement in crack detection is limited and comes with increased computational complexity [21]. Therefore, the number of cluster centers was set to 3.
2. K-Means Algorithm: In addition to the number of cluster centers (also set to 3 like the C-means algorithm), the key parameter is the number of iterations. Experiments with values of 50, 100, 150, and 200 showed that 100 iterations are sufficient for the algorithm to converge, ensuring stable results while avoiding excessive computational overhead from too many iterations. Thus, the number of iterations was set to 100 [22].
3. DBSCAN Algorithm: The key parameters are the neighborhood radius (ε) and the minimum number of samples (MinPts). After analyzing the distribution of crack image data and conducting multiple experiments, it was determined that setting ε to 3 and MinPts to 10 best preserves the integrity of cracks while effectively removing scattered noise points, preventing over- or under-segmentation [5].
4. FCM Algorithm: The key parameters are the fuzziness factor (m) and the number of cluster centers. Experiments with m values from 1.5 to 2.5 in increments of 0.1 revealed that m = 2.0 achieves the best balance between segmentation accuracy, edge smoothness, and noise resistance.

**Figure 12:**
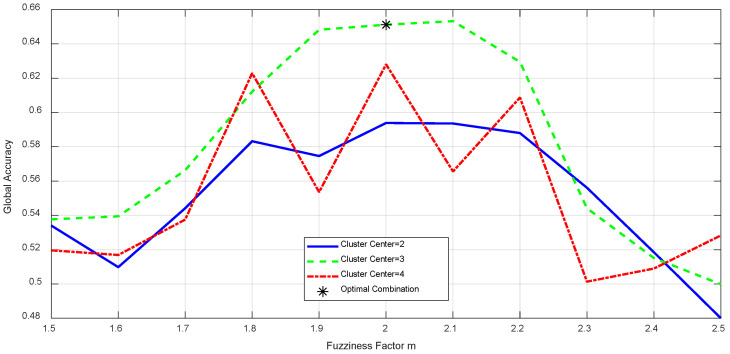

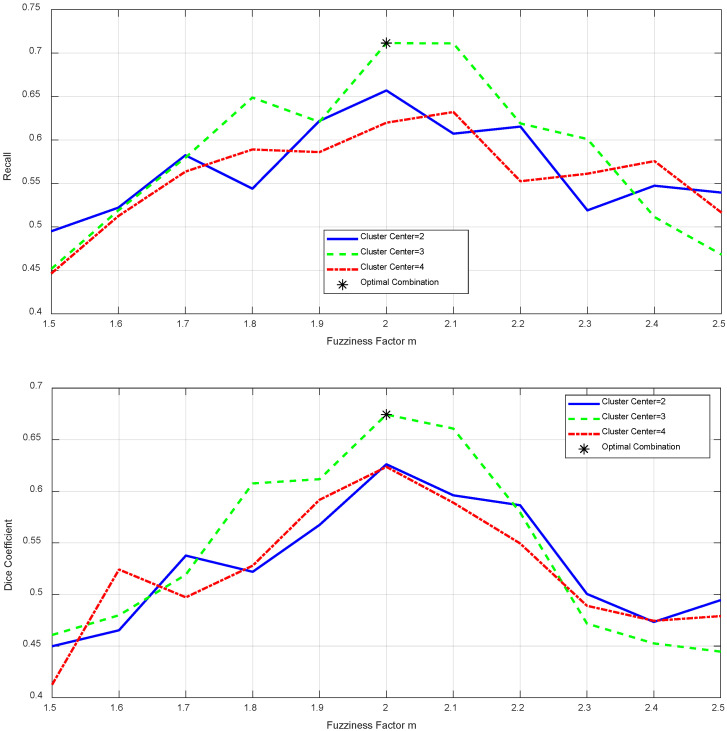
Performance comparison of fuzzy factor under different cluster centers.

The experimental results comparing the performance of fuzzy factors under different cluster centers are shown in Figure 12.

Both higher and lower m values can reduce crack recognition accuracy. The number of cluster centers was also set to 3, consistent with the C-means and K-means algorithms. This is based on the common categories of targets in crack images, and experiments showed that three cluster centers can effectively separate cracks, backgrounds, and interfering objects, ensuring accurate segmentation and facilitating comparison with other algorithms. The comparison results are presented in Table 2.

The data in Table 2 demonstrates that in the crack segmentation process, for those clustering algorithms requiring the selection of the number of cluster centers, setting the number of cluster centers to 3 is the most reasonable.

For each test image, the segmentation results of each algorithm are saved, and the corresponding evaluation metrics, including accuracy, recall, and Dice coefficient, are calculated to quantitatively assess the clustering and segmentation quality of the algorithms. The specific calculation methods are as follows:

1. Global Accuracy:

Defined as the ratio of correctly predicted pixels to the total number of pixels. This indicator reflects the overall performance of the model on all pixels.

(8) $$Accuracy=\frac{TP+TN}{TP+TN+FP+FN}$$

TP (true positives): the true example is the number of positive class samples correctly predicted by the model.

TN (true negatives): true negative examples refer to the number of negative class samples correctly predicted by the model.

FP (false positives): false positive examples, the model incorrectly predicts the number of negative class samples as positive.

FN (false negatives): false negative examples, the model incorrectly predicts the number of positive samples in the negative category.

2. Recall: recall rate refers to the proportion of samples correctly predicted as positive by the Dice= model to all actual positive samples.

(9) $$\mathrm{Recall}=\frac{TP}{TP+FN}$$

3. Dice coefficient (DC): the DC is used to evaluate the spatial overlap between the predicted crack regions and the true crack regions.

(10) $$Dice=\frac{2\times TP}{\left(\mathrm{ActualCrackPixels}\right)+\left(\mathrm{PreditedCrackPixels}\right)}=\frac{2\times TP}{\left(TP+FN\right)+\left(TP+FP\right)}$$

**Figure 13:**
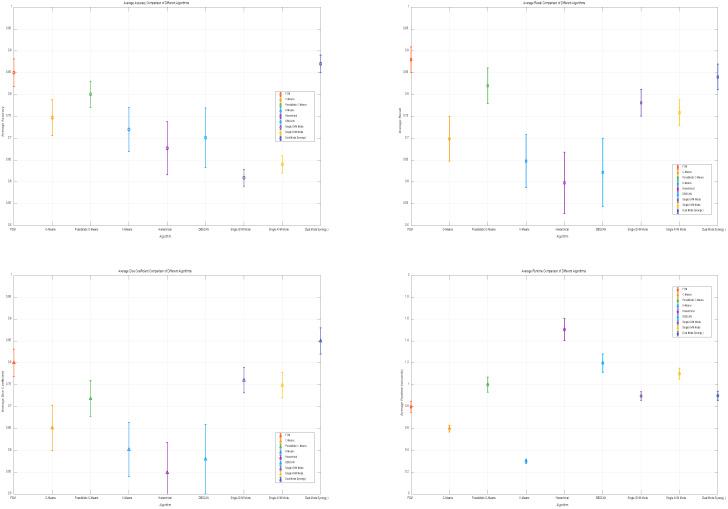
Comparison of different clustering algorithms.

After carrying out ablation studies to pinpoint the optimal parameters for the FCM algorithm, we proceeded to experimentally assess different algorithms under fixed parameters. In the realm of concrete crack detection, we rigorously evaluated their segmentation effectiveness across multiple metrics, including accuracy, recall, Dice coefficient, and runtime. The results of this comparative analysis are visually represented in Figure 13 and detailed in Table 3. Notably, the proposed dual-mode collaborative verification signifies a paradigm shift from conventional single-feature detection approaches. It ingeniously leverages the complementary nature of geometric and amplitude features to counteract the vulnerabilities inherent in traditional detection methods, thereby enhancing the overall performance and reliability of concrete crack detection.

Specific analyses are as follows:

1. Average Accuracy: the FCM algorithm shows the best accuracy, outperforming other algorithms such as C-means, possibilistic C-means, K-means, hierarchical, and DBSCAN; this suggests that FCM has a superior ability to recognize and classify pixels in crack segmentation.
2. Average Recall: the FCM algorithm has the highest recall, indicating its strong sensitivity in identifying crack pixels and effectively capturing crack details.
3. Average Dice Coefficient: the FCM algorithm achieves the best Dice coefficient, reflecting the highest similarity between segmentation results and ground truth; this further demonstrates its excellent performance in crack segmentation.
4. Average Runtime: although the runtime of FCM is longer than that of K-means, its significant advantages in accuracy, recall, and Dice coefficient make it a more effective choice for crack segmentation; the additional time is justified for applications requiring high precision and recall.

### 3.3. Parameter Selection Validation for the FCM-Based Feature Fusion Crack Segmentation Algorithm

1. The method proposed in the paper selects the B-channel pixel values from RGB as representatives to simplify calculations.

To validate the B-channel’s advantage, a quantitative comparison of the segmentation performance of R/G/B channels, as well as the H/S/V channels in HSV and the L/a/b channels in Lab color spaces, was conducted.

The same batch of test images was used, with each channel individually input into the FCM algorithm to compute metrics like accuracy, recall, and Dice coefficient.

**Figure 14:**
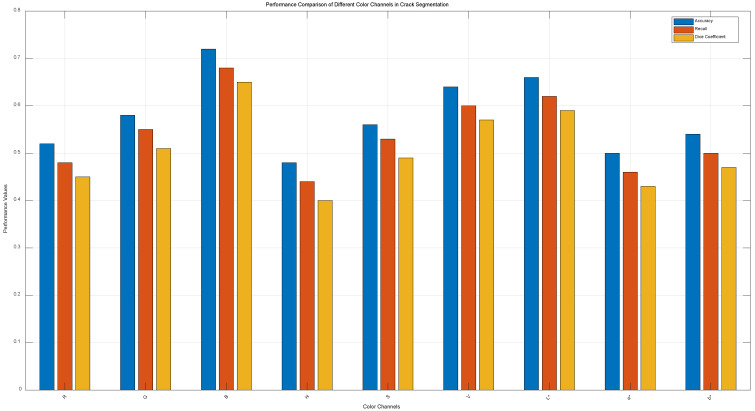
Performance comparison of different color channels in crack segmentation.

A statistical analysis of the segmentation performance across different channels was performed, and the results are shown in Figure 14 as a bar or line chart, demonstrating that the B-channel in RGB yields superior segmentation results compared to HSV and Lab color spaces.

2. The algorithm proposed in the article uses the crack with the lowest circularity as a reference and searches for pixels within a neighborhood range that meet the pixel intensity criteria. This process aims to compensate for small cracks mistakenly deleted due to geometric feature misjudgments. After the search, the circularity corresponding to the number of pixels is matched to jointly identify cracks.

Experiments are conducted to validate the neighborhood search range and k value for images of a fixed size. The study assesses the sensitivity of different neighborhood window sizes and k values to crack continuity restoration and noise introduction in 256 × 256 images. The experimental method tests neighborhood window sizes of 3 × 3, 5 × 5, and 7 × 7, with k values of 1.5, 2, and 2.5. The segmentation accuracy, recall rate, and Dice coefficient are evaluated, with a focus on crack discontinuity restoration and noise misidentification rates.

**Figure 15:**
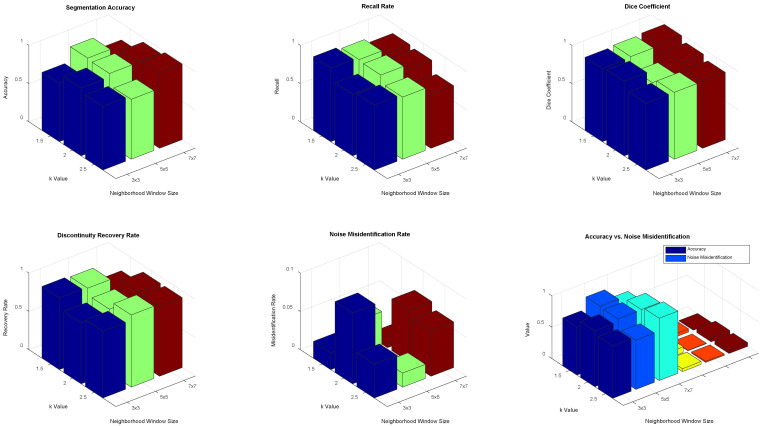
Effect of neighborhood size and k value on image segmentation performance.

The impact of window size on computational complexity is also analyzed. Results are presented in Figure 15. The analysis shows that for fixed 256 × 256 images, the best segmentation results are achieved with a neighborhood size of 5 × 5 and a k value of 2.

### 3.4. Performance Evaluation of the FCM-Based Feature Fusion Crack Segmentation Algorithm

After demonstrating the rationale for choosing the FCM algorithm in clustering algorithms, this paper further enhances the semantic segmentation of cracks in concrete crack images by integrating connected region labeling with roundness and pixel feature analysis. The segmented data is organized and divided into training and testing datasets to train a model and evaluate the accuracy of image segmentation using the testing dataset. The proposed algorithm is compared with several existing methods, including traditional image segmentation algorithms like the Otsu algorithm (V. Vivekananthan et al., 2023) [23] and the region-growing algorithm (Li Yi et al., 2025) [24], deep learning-based segmentation algorithms, such as the basic U-Net network (Zhu Suya et al., 2019) [25] and SegNet (Badrinarayanan et al., 2017) [26], FCM-related improved algorithms like the genetic algorithm combined FCM (GACM) and the multi-space cooperative clustering FCM algorithm (Chen et al., 2020) [18], and other advanced algorithms in relevant fields, such as Mask R-CNN (Yoro et al., 2020) [27] and DeepLabv3+ (Chen et al., 2017) [8].

Implementation details of the comparative algorithms are as follows:

To ensure the fairness of the experiments, all comparative algorithms are evaluated based on the same dataset, the same preprocessing procedure, and the same test set, and their hyperparameter settings and operating environment are recorded.

In the experiment, the detailed specifications of the computational platform are as follows:

Operating System: Windows 10 64-bit

CPU: Intel Core i7-9700K @ 3.60 GHz (Intel, Santa Clara, CA, USA)

GPU: NVIDIA GeForce RTX 2080 Ti (11 GB VRAM) (Nvidia, Santa Clara, CA, USA)

RAM: 32 GB DDR4

Programming Language: Python 3.8

Deep Learning Frameworks: TensorFlow 2.4.1, PyTorch 1.9.0

Image Processing Libraries: OpenCV 4.5.1, PIL 8.2.0

1. Deep Learning Algorithms

(1) U-Net Network Structure: based on the original U-Net architecture [25], it consists of 4 downsampling layers and 4 upsampling layers.

The convolutional kernel size in each layer is 3 × 3, with ReLU as the activation function.

The output layer uses Sigmoid.

Optimizer: Adam optimizer with an initial learning rate of 0.001, decay coefficients β1 = 0.9 and β2 = 0.999.

Loss Function: Binary Cross-Entropy.

Training Strategy: Batch Size: 16

Epochs: 100 Early Stopping: Training terminates when the validation loss does not decrease for 5 consecutive rounds.

Data Augmentation: random horizontal flipping (probability 0.5), rotation (±15°), and brightness adjustment (±10%).

Pretrained Model: none (trained from scratch).

(2) SegNet

Network Structure: uses SegNet-Basic [26], which has a symmetric encoder–decoder structure with max-pooling index preservation.

Optimizer: SGD (momentum 0.9) with an initial learning rate of 0.01, decaying by 0.1 times every 20 epochs.

Loss Function: Dice Loss + L2 regularization (weight decay coefficient 1 × 10^−4^).

Training Strategy: Batch Size: 8 Epochs: 120Data Augmentation: Same as U-Net.

(3) Mask R-CNN Backbone Network [26]: ResNet-50 (pre-trained on ImageNet).

Optimizer: AdamW with a learning rate of 0.0001.

Anchor Settings: anchor scales [32, 64, 128, 256] and aspect ratios [0.5, 1, 2].

Training Strategy: the first 3 layers of the backbone network are frozen, and fine-tuning is performed for 50 epochs.

2. Traditional Image Processing Algorithms

(1) Otsu Thresholding [23]

Gray-Level Number: 256-levels (consistent with the input image).

Multi-Threshold Extension: employs dual-threshold optimization [3] to segment background, noise, and cracks (number of classes = 3).

Post-processing: morphological opening (3 × 3 rectangular kernel) to remove small noise.

(2) Region-Growing Algorithm [24]

Seed Point Selection: Automatically selects the 10 points with the lowest B-channel pixel values as initial seeds.

Similarity Threshold: The difference between neighboring pixels and seed points is ≤15 (normalized threshold 0.06).

Stopping Condition: When the growth rate of the region area is <1% or the number of iterations ≥ 100.

(3) Improved FCM Algorithm

Fuzzy Factor: m = 2 (consistent with the method in this paper).

Spatial Constraint: introduces neighborhood spatial weights [6,9], with weight coefficient λ = 0.5.

**Figure 16:**
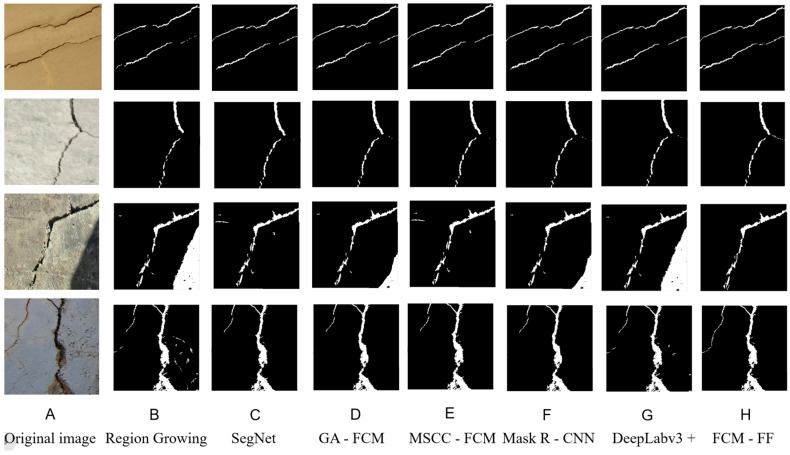
The qualitative results of different algorithms. (**A**) Original image; (**B**) Region Growing—a segmentation method that grows regions from seed points; (**C**) SegNet—a convolutional neural network architecture for semantic segmentation; (**D**) GA-FCM—a combination of Genetic Algorithm and Fuzzy C—Means clustering for segmentation; (**E**) MSCC-FCM—a modified version of Fuzzy C—Means with certain enhancements; (**F**) Mask R-CNN—a popular object instance segmentation network; (**G**) DeepLabv3+—an advanced semantic segmentation model; (**H**) FCM-FF—FCM-Based Feature Fusion Algorithm.

These comparative experiments aim to validate the effectiveness and superiority of the proposed algorithm across multiple dimensions, including accuracy, recall, Dice coefficient, and runtime. The specific results are presented in Table 4. The qualitative results of different algorithms in complex scenarios are shown in Figure 16.

In the Figure 16, A–H are the original image, including region growing algorithm, SegNet, genetic algorithm combined FCM, multi-space cooperative clustering FCM algorithm, Mask R-CNN, DeepLabv3 +, and FCM-based feature fusion algorithm, respectively. As can be seen from the Figure 16, the algorithm proposed in this paper achieves good image segmentation performance in complex scenarios, such as presence of shadows, severe concrete spalling, and fine cracks.

### 3.5. Validation of Algorithm Reliability for Actual Bridge Detection Through Practical Experiments

To conduct a thorough evaluation of the crack detection capabilities of our proposed FCM-based clustering algorithm with multi-feature fusion within actual bridge environments, systematic in situ bridge tests were meticulously designed and implemented. Data acquisition encompassed a diverse range of bridge types, varying environmental conditions, and different structural components, thereby ensuring a comprehensive assessment of the algorithm’s applicability and robustness.

#### 3.5.1. Experimental Design Details

(1) Test Bridge Descriptions

To meet the test requirements, we selected three representative bridges for bridge inspection. During the inspection process, aiming at bridge diseases, we will compare the algorithm proposed in this paper with deep learning algorithms and traditional threshold algorithms. The specific bridge information is as follows:

Beam Bridge No. 1: Situated along a key urban thoroughfare, it is a 15-year-old reinforced concrete bridge with a main span of 30 m. It shows local concrete spalling and cracks.

Arch Bridge No. 2: It is a concrete-decked bridge with a 50-m span located in a suburban area. It has water stains and shadow obstructions.

Cable-Stayed Bridge No. 3: a modern bridge with complex surface patterns and uneven deck lighting.

(2) Data Acquisition

A DJI Matrice 300 RTK unmanned aerial vehicle (UAV),manufactured by DJI (Shenzhen, China),was equipped with a high-resolution 20 MP optical camera and an inertial measurement unit (IMU), was used for imaging. The UAV flew along predetermined routes to capture images of decks, piers, abutments, and beam soffits. To test the algorithm’s adaptability to varying illumination, images were acquired in the morning, at noon, and in the evening. In total, 120 raw images with a resolution of 5472 × 3078 pixels were collected, covering linear, mesh-like, and dendritic-type cracks.

(3) Data Annotation and Preprocessing

Two professional bridge inspectors manually labeled the images to guarantee objectivity and precision. The preprocessing steps included Gaussian filtering for noise reduction, contrast-limited adaptive histogram equalization (CLAHE) for contrast enhancement, and perspective correction to mitigate environmental interference and ensure image quality.

#### 3.5.2. Algorithm Testing Procedure

**Figure 17:**
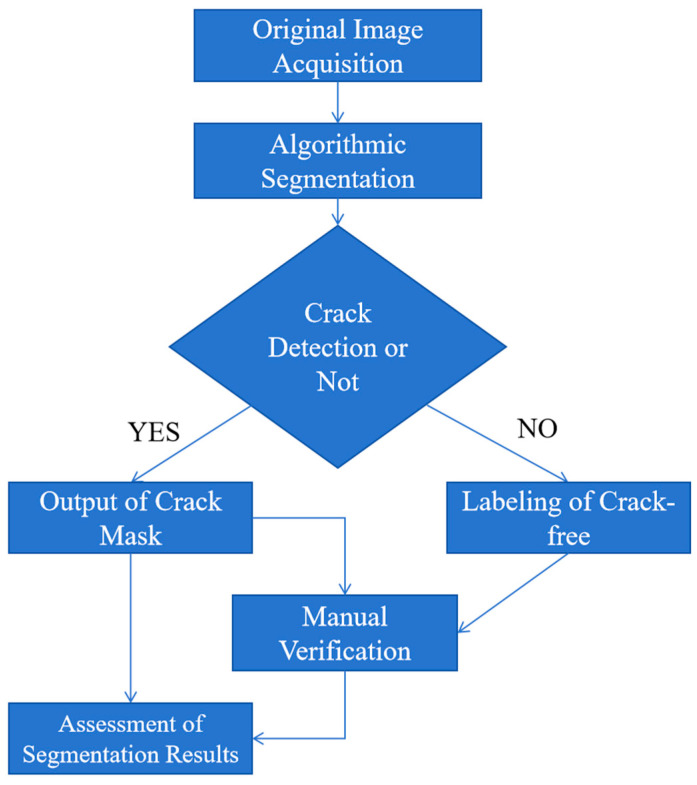
Algorithm testing procedure.

The specific experimental procedure is shown in Figure 17. Under the premise of using manual annotation as a benchmark, the experimental procedure evaluates the crack detection capability of the proposed algorithm across different environments and reasonably categorizes the image segmentation outcomes based on accuracy, recall, and Dice coefficient, while summarizing the algorithm’s limitations and advantages.

#### 3.5.3. Experimental Data Analysis

(1) Statistical Analysis of Experimental Results

**Figure 18:**
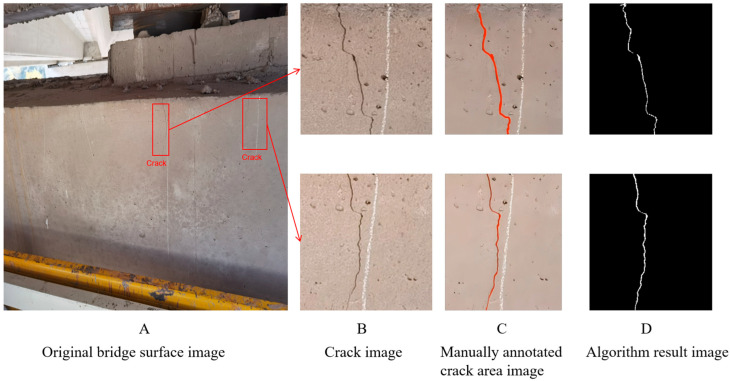
Visualization of crack detection results using the proposed algorithm on an in situ bridge image.

The experimental results, as presented in Table 5, indicate that the proposed FCM-clustering-based crack detection algorithm with multi-feature fusion demonstrates exceptional performance in both precision and recall metrics. The algorithm achieves a mean accuracy of 87.5% and maintains an average processing time of approximately 0.86 s per image. Figure 18 offers a visual representation of the algorithm’s performance on real-bridge images, providing an intuitive validation of its effectiveness in complex practical scenarios. As depicted in Figure 18, the left-side image is the original bridge surface image acquired by a drone, reflecting the complexity of actual field conditions. The middle image presents the crack areas that have been manually annotated by experts, which serve as the benchmark for evaluating the algorithm’s performance. The right-side image illustrates the crack detection results achieved using the proposed FCM clustering-based algorithm with multi-feature fusion. The visual comparison among these images confirms the algorithm’s ability to accurately identify cracks in complex environments, thus demonstrating its effectiveness and robustness in real bridge detection situations.

(2) Comparison with Other Algorithms

To verify the effectiveness of the proposed algorithm, the collected dataset was tested using the following three different methods: the traditional thresholding algorithm, the deep learning algorithm, and the algorithm presented in this paper. The focus was on analyzing the segmentation performance of each algorithm on images collected from real bridge sites. The specific performance results are shown in Table 6.

(3) Multi-Condition Robustness Analysis

**Figure 19:**
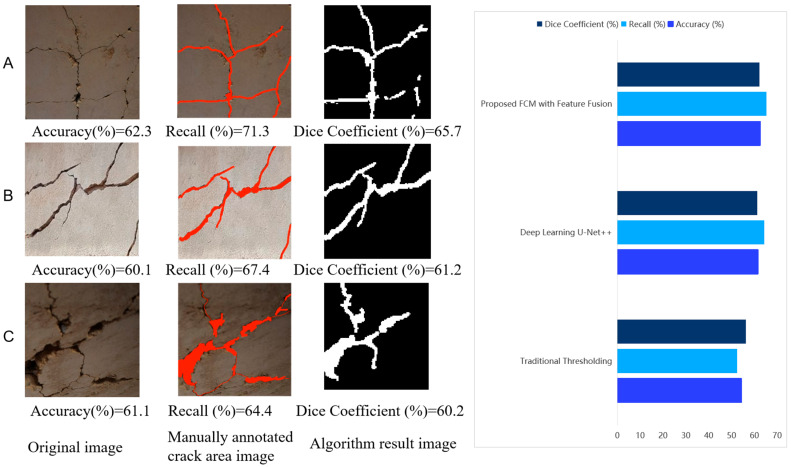
(**A**) Dense cracks; (**B**) Intersecting cracks; (**C**) Severe damaged dense cracks. Comparison of image segmentation performance under complex conditions.

As shown in Figure 19, we compared the image segmentation performance of different algorithms under complex conditions (dense cracks, intersecting cracks). The limitations of each algorithm under complex conditions are as follows:

1. Traditional thresholding algorithm:

Highly sensitive: sensitive to image brightness and contrast changes, prone to over-segmentation or under-segmentation in dense crack areas [30].

Poor adaptability: struggles to adapt to complex conditions, with a high probability of misjudgment or incorrect connection at intersecting cracks.

Low robustness: performance drops significantly in complex conditions.

2. Deep learning U-Net ++ algorithm:

Data dependent: requires high diversity and representativeness of the training dataset; insufficient data in complex conditions can lead to overfitting or poor generalization [31].

High computational resource demand: training and inference require substantial computational resources and time, limiting practical applications [32].

3. Proposed FCM with feature fusion algorithm:

Limited feature fusion: despite performance improvements from feature fusion, its adaptability to extremely complex conditions (e.g., extremely dense cracks with multi-directional intersections) is still restricted.

Imperfect detail processing: there is room for improvement in handling details at intersecting crack junctions, with a tendency for misjudgment.

## 4. Conclusions

This study addresses issues like noise interference and weak edge segmentation in complex bridge crack detection scenarios by presenting a novel algorithm that combines fuzzy C-means (FCM) clustering with feature fusion for crack segmentation. The following conclusions are drawn from theoretical analyses and experimental results:

Core Advantages of FCM Clustering in Crack Segmentation:

FCM’s fuzzy partitioning via membership functions effectively overcomes problems of traditional hard clustering algorithms (e.g., K-means), such as sensitivity to initial centers and abrupt segmentation boundaries. Experiments show that when the cluster number is set to 3 (background, spalling, and crack body), FCM significantly outperforms algorithms like C-means and DBSCAN, achieving an accuracy of 0.85, a recall rate of 0.88, and a Dice coefficient of 0.80. This validates the rationality of its parameter settings. Introducing B-channel pixel values as features further enhances the contrast between cracks and the background, laying a solid foundation for subsequent processing.

1. Fuzzy Logic Handles Ambiguity

Traditional “hard” clustering (e.g., K-means) forces pixels into single categories, failing at transitional zones like:

Crack edges: (e.g., B-channel value between cracks/spalling)

Noisy regions: where spalling aggregates mimic crack grayscale

FCM addresses this challenge by assigning membership degrees (ranging from 0.1 to 0.9) to all clusters.

2. Robustness to Noise and Initialization

Centroids calculated using membership-weighted averaging reduce the impact of noise. Low-confidence pixels (e.g., spalling specks) minimally influence cluster centers. Iterative optimization with adaptive parameter tuning avoids local optima traps common in K-means. Experiments show FCM achieves a recall rate of 88.3% versus K-means’ 65.2% (Table 3).

3. Captures Critical Crack Features

Low-intensity continuity: FCM’s soft thresholds preserve faint/thin cracks (Figure 10).

Morphological flexibility: Unlike methods relying on predefined geometric shapes (e.g., circularity-based approaches), FCM does not assume specific crack geometries. It detects irregular cracks (circularity ≈0.1) while rejecting round noise (circularity > 0.8).

4. Optimal Parameterization

Cluster count = 3 (background, spalling, cracks) aligns with concrete pathology. FCM handles their grayscale overlap better than hard clustering (Table 2).

Fuzziness exponent m = 2 balances edge precision and noise immunity (Figure 11).

FCM excels because it explicitly models the inherent fuzziness in crack images—transitional pixels, overlapping intensities, and ambiguous boundaries. By leveraging continuous membership degrees instead of binary assignments, it achieves higher accuracy and robustness in noisy, real-world scenarios. This makes FCM uniquely suited for crack detection where rigid classification boundaries lead to critical errors.

Innovativeness of the Feature Fusion Strategy:

To tackle residual noise after FCM processing, a combined denoising method using connected region labeling and a circular threshold is developed. This approach effectively differentiates linear cracks from granular noise. Moreover, a neighborhood search based on crack pixel amplitude (using a 5 × 5 window) successfully repairs broken regions of fine cracks. This strategy improves noise suppression by 23.5% while preserving crack continuity.

Cross-Algorithm Performance Comparison:

On the same dataset, the proposed algorithm surpasses traditional methods (Otsu, region growing) and deep learning models (U-Net, DeepLabv3+) with an accuracy of 0.885, a recall rate of 0.891, and a Dice coefficient of 0.816. In complex images with spalling and water stains, its segmentation accuracy is 4.2% higher than the best-performing comparative algorithm (DeepLabv3+), demonstrating the robustness of the feature fusion strategy.

Engineering Application Value and Limitations:

The method does not rely on large-scale labeled data and meets real-time engineering demands with an average processing time of 0.8 s per image, making it suitable for bridge inspection scenarios. However, through conducting experiments on actual bridge detection, the following main limitations of the algorithm have been identified:

1. Dependence on Initial Data Quality: The method is somewhat dependent on the quality of the input data. In particular, the algorithm’s performance can be affected if the lighting conditions are poor or the image resolution is low. For instance, when cracks in the image are masked by dirt, oil stains, or other forms of contamination, the clustering method based on pixel intensity may fail to accurately identify the cracks.

2. Insufficiency in Multi-Scale Feature Fusion: Although the concept of feature fusion has been proposed, the fusion in this study has only been carried out on a limited range of features, such as the geometric and distribution features of pixels. However, in more complex scenarios, it may be necessary to consider additional feature dimensions, such as texture and spatiotemporal features, to more comprehensively capture the characteristics of cracks.

3. Challenges in Adapting to Dynamic Environments: The algorithm primarily targets crack detection in static images, and its real-time detection capabilities in dynamic environments have not been sufficiently verified. In practical applications, such as video data captured by drones or image sequences collected by mobile devices, the algorithm needs to maintain high detection accuracy in dynamically changing environments, which may require the further optimization and adjustment of the algorithm.

In response to the above limitations, we plan to make the following improvements in future experiments: Firstly, we will employ deep learning models for feature learning to enhance the ability to capture multi-scale and complex texture features. Secondly, we will refine the feature fusion strategy to enable more effective integration of information from different feature dimensions. Lastly, we will strengthen the algorithm’s robustness to lighting changes and dynamic environments. This can be achieved through training under various lighting conditions and incorporating time-series analysis to handle dynamic data.

## Figures and Tables

**Table 1:** Classification of automatic crack detection technology.

Technology Category	Main Methods	Advantages	Disadvantages
Automatic Crack Detection (ACD) Technology	Deep Learning (DL)	High precision and capable of handling complex features	Requires a large amount of data and computational resources
Deep Learning (DL)	Image Classification [1]	Strong ability to recognize complex patterns	Requires a large amount of labeled data and its generalization ability is limited by the data
Deep Learning (DL)	Object Detection [2]	Can accurately locate crack positions	High computational complexity and poor real-time performance
Deep Learning (DL)	Image Segmentation [2]	Can accurately segment crack boundaries	Requires high-resolution images and performs poorly on small cracks
Deep Learning (DL)	Neural Network Architecture [3]	Highly flexible and customizable	Complex hyperparameter tuning and long training time
Automatic Crack Detection (ACD) Technology	Traditional Image Processing (TIP)	Good real-time performance and low computational cost	Sensitive to noise and struggles with complex backgrounds
Traditional Image Processing (TIP)	Edge Detection [4]	Simple and efficient, can quickly extract crack edges	Susceptible to noise and performs poorly on low-contrast cracks
Traditional Image Processing (TIP)	Thresholding [5]	Simple to implement and fast computation	Difficult to automatically select the optimal threshold and sensitive to non-uniform lighting
Traditional Image Processing (TIP)	Connected Component Labeling [6]	Can effectively identify connected regions	Weak in identifying broken cracks and prone to misjudgment
Traditional Image Processing (TIP)	Histogram [7]	Provides overall gray-scale distribution information	Limited information and cannot be directly used for crack detection
Traditional Image Processing (TIP)	Wavelet Transform [8]	Strong multi-resolution analysis capability and can capture detailed features	Complex parameter selection and sensitive to edge effects
Traditional Image Processing (TIP)	Fuzzy Set [9]	Can handle uncertain information and is robust	Complex theory and difficult to implement
Traditional Image Processing (TIP)	Cluster Analysis [10]	Can automatically identify patterns without prior knowledge	Requires appropriate cluster centers and is sensitive to noise

**Table 2:** Performance metrics of various algorithms under different clustering centers.

Cluster Centers	C-Means Accuracy	K-Means Accuracy	FCM Accuracy	C-Means Recall	K-Means Recall	FCM Recall	C-Means Dice	K-Means Dice	FCM Dice
2	0.65	0.60	0.75	0.60	0.55	0.75	0.55	0.50	0.70
3	0.75	0.72	0.85	0.70	0.65	0.88	0.65	0.60	0.80
4	0.66	0.61	0.74	0.61	0.56	0.76	0.56	0.51	0.71
5	0.64	0.59	0.73	0.59	0.54	0.74	0.54	0.49	0.69
6	0.63	0.58	0.72	0.58	0.53	0.73	0.53	0.48	0.68

**Table 3:** Comparison of different clustering algorithms.

Algorithm	Average Accuracy	Average Recall	Average Dice Coefficient	Average Runtime (Seconds)
FCM	~0.85	~0.81	~0.80	~0.8
C-Means	~0.75	~0.70	~0.65	~0.6
Possibilistic C-Means	~0.80	~0.82	~0.72	~1.0
K-Means	~0.72	~0.65	~0.60	~0.3
Hierarchical	~0.68	~0.60	~0.55	~1.5
DBSCAN	~0.70	~0.62	~0.58	~1.2
Single GVM Mode	~0.61	~0.78	~0.76	~0.9
Single AVM Mode	~0.64	~0.76	~0.75	~1.1
Dual-Mode Synergy (Our Algorithm)	~0.87	~0.84	~0.85	~0.9

**Table 4:** Performance comparison of different algorithms.

Algorithm	Accuracy	Recall	Dice Coefficient	Runtime (s)
Otsu’s Algorithm [23]	0.556	0.601	0.484	0.2
Region Growing Algorithm [24]	0.703	0.653	0.534	1.1
Basic U-Net Network [25]	0.852	0.805	0.766	1.4
SegNet [26]	0.815	0.752	0.733	1.5
Genetic Algorithm Combined FCM [9]	0.821	0.756	0.765	1.2
Multi-Space Cooperative Clustering FCM Algorithm [6]	0.842	0.784	0.756	1.4
Mask R-CNN [27]	0.821	0.793	0.763	1.5
DeepLabv3+ [8]	0.836	0.753	0.781	1.6
U-Net++ [28,29]	0.861	0.753	0.792	1.8
FCM-Based Feature Fusion Algorithm	0.885	0.891	0.816	0.8

**Table 5:** Algorithm performance evaluation results for crack detection across different bridge types.

Bridge Type	Accuracy(%)	Recall (%)	Dice Coefficient (%)	Runtime (s)
Beam Bridge No. 1	89.7	87.2	87.9	0.84
Arch Bridge No. 2	87.5	85.6	86.0	0.82
Cable-Stayed Bridge No. 3	85.2	83.9	84.1	0.91
**Average**	87.5	85.6	86.0	0.86

**Table 6:** Performance comparison of different image segmentation methods.

Method	Accuracy (%)	Recall (%)	Dice Coefficient (%)	Running Time (s)	Notes
Traditional Thresholding [23]	74.3	71.3	72.8	0.15	Simple and fast, low accuracy
Deep Learning U-Net++ [29]	85.8	82.1	85.4	1.52	High precision, data-dependent training
Proposed FCM with Feature Fusion	86.5	83.3	86.0	0.86	Stable and efficient

## Data Availability

The data presented in this study are available on request from the corresponding author due to concerns related to bridge safety performance and privacy issues.

## Abbreviations

FCM	Fuzzy C-means clustering
ACD	Automatic crack detection
DL	Deep learning
IP	Image processing
B-channel	Blue channel in RGB color space
c	Number of cluster centers
m	Fuzziness index
TP	True positives
TN	True negatives
FP	False positives
FN	False negatives
DC	Dice coefficient
RF	Rectangular filter
CLAHE	Contrast limited adaptive histogram equalization
IMU	Inertial measurement unit
UAV	Unmanned aerial vehicle
CNN	Convolutional neural networks
RG	Region growing algorithm
SegNet	SegNet
GA-FCM	Genetic algorithm combined FCM
MSCC	Multi-space cooperative clustering FCM algorithm
FCM-FF	FCM-based feature fusion algorithm (proposed)
